# Prevalence of Non-alcoholic Fatty Liver Disease in Patients With Inflammatory Bowel Disease: A Systematic Review and Meta-Analysis

**DOI:** 10.7759/cureus.97094

**Published:** 2025-11-17

**Authors:** Anastasia Postoev, Abebe Yigzaw, Helai Hussaini, Olaniyi Fadeyi, Rahman Hameed Mohammed Abdul, Sonalben Chaudhary, Mohammed Qasim Rauf, Neelum Ali

**Affiliations:** 1 Internal Medicine, Caribbean Medical University, Willemstad, CUW; 2 Internal Medicine, Addis Ababa University School of Medicine, Addis Ababa, ETH; 3 Internal Medicine, West Anaheim Medical Center, Anaheim, USA; 4 Gastroenterology and Hepatology, Royal Derby Hospital, Stoke-on-Trent , GBR; 5 Internal Medicine, Zydus Sitapur Hospital, Sitapur, IND; 6 Trauma and Orthopaedics, The Hillingdon Hospitals National Health Trust (NHS) Foundation Trust, London, GBR; 7 Internal Medicine, University of Health Sciences, Lahore, PAK

**Keywords:** gut-liver axis, inflammatory bowel disease, meta-analysis, non-alcoholic fatty liver disease, prevalence

## Abstract

Non-alcoholic fatty liver disease (NAFLD) has emerged as one of the significant comorbidities in patients with inflammatory bowel disease (IBD). This systematic review and meta-analysis aimed to synthesize existing evidence on NAFLD prevalence in IBD patients and explore sources of heterogeneity. A comprehensive literature search was conducted across PubMed/MEDLINE, Embase, Web of Science, and Scopus from January 2016 to September 2025, including studies reporting NAFLD prevalence in adult IBD patients diagnosed through imaging, biopsy, or validated biomarkers. Two independent reviewers screened studies and extracted data on patient demographics, IBD subtypes, and NAFLD diagnostic methods. Quality assessment was performed using the Newcastle-Ottawa Scale. Thirty-five studies encompassing over 47 million IBD patients were included. The pooled prevalence of NAFLD in IBD patients was 26% (95% CI: 23-29%), with substantial heterogeneity (I² = 99.9%). Subgroup analysis revealed a higher prevalence in cross-sectional studies (40.9%) compared to retrospective studies (19.5%). Crohn's disease patients demonstrated higher NAFLD prevalence (26.1%) than ulcerative colitis patients (17.0%). Studies published from 2020 onwards reported a slightly higher prevalence (27.5%) compared to earlier studies (24.8%). The findings indicate that approximately one in four IBD patients has concurrent NAFLD, representing a substantial comorbidity burden. These results underscore the importance of systematic NAFLD screening in IBD patients and integrated multidisciplinary care addressing both gastrointestinal and metabolic complications.

## Introduction and background

Non-alcoholic fatty liver disease (NAFLD) has emerged as the most prevalent chronic liver condition worldwide, affecting approximately 25% of the global population [1]. The condition encompasses a spectrum of hepatic pathology ranging from simple steatosis to non-alcoholic steatohepatitis (NASH), which can progress to cirrhosis and hepatocellular carcinoma [2]. Concurrently, inflammatory bowel disease (IBD), comprising Crohn's disease and ulcerative colitis, represents a chronic inflammatory condition affecting millions globally, with increasing incidence rates particularly in newly industrialized countries [3]. The intersection of these two conditions has garnered considerable attention in recent years, as accumulating evidence suggests a higher prevalence of NAFLD among IBD patients compared to the general population [4].

The pathophysiological link between IBD and NAFLD remains incompletely understood but appears to be multifactorial. Chronic systemic inflammation, characteristic of IBD, may contribute to hepatic lipid accumulation through inflammatory cytokines such as tumor necrosis factor-alpha and interleukin-6, which interfere with insulin signaling and promote hepatic steatosis [5]. Additionally, gut dysbiosis in IBD patients may increase intestinal permeability, allowing bacterial products to reach the liver via the portal circulation, potentially triggering hepatic inflammation and steatosis [6]. Furthermore, certain medications commonly used in IBD management, including corticosteroids and methotrexate, have been associated with hepatic steatosis, though their contribution to the overall NAFLD burden remains debated [7].

The clinical significance of NAFLD in IBD patients extends beyond hepatic complications. NAFLD may influence IBD disease activity, treatment response, and overall prognosis [8]. Moreover, the presence of metabolic syndrome components, frequently observed in NAFLD patients, can complicate IBD management and increase cardiovascular risk [9]. Despite growing recognition of this association, reported prevalence rates of NAFLD in IBD populations vary considerably across studies, ranging from 1.5% to over 40%, depending on diagnostic methods, geographical location, and patient populations studied [10,11].

Current diagnostic approaches for NAFLD include imaging modalities such as ultrasound, computed tomography, and magnetic resonance imaging, as well as histological examination through liver biopsy, which remains the gold standard but is limited by its invasive nature [12]. Non-invasive biomarkers and scoring systems have been developed but require validation in IBD populations [13]. The heterogeneity in diagnostic criteria and assessment methods contributes to the variability in reported prevalence rates, necessitating a comprehensive synthesis of available evidence.

Understanding the true prevalence of NAFLD in IBD patients is crucial for several reasons. First, it informs screening strategies and clinical surveillance protocols. Second, it guides therapeutic decision-making, particularly regarding medications with potential hepatotoxic effects. Third, it highlights the need for integrated multidisciplinary care addressing both gastrointestinal and metabolic complications [14]. This systematic review and meta-analysis aim to synthesize existing evidence regarding the prevalence of NAFLD in IBD patients, explore sources of heterogeneity, and identify knowledge gaps requiring future investigation.

## Review

Methodology

Literature Search

The study was reported in accordance with the Preferred Reporting for Systematic Reviews and Meta-Analysis (PRISMA) guidelines [15]. A comprehensive systematic literature search was conducted across multiple electronic databases, including PubMed/MEDLINE, Embase, Web of Science, and Scopus, from January 1, 2016, to September 20, 2025. The search strategy was developed by combining Medical Subject Headings (MeSH) terms and free-text words related to NAFLD and IBD. The following search terms were used in various combinations using Boolean operators (AND, OR): "non-alcoholic fatty liver disease" OR "NAFLD" OR "non-alcoholic steatohepatitis" OR "NASH" OR "hepatic steatosis" OR "fatty liver" AND "inflammatory bowel disease" OR "IBD" OR "Crohn's disease" OR "ulcerative colitis" OR "UC" OR "CD". The search was limited to human studies with no language restrictions. Additionally, reference lists of included studies and relevant review articles were manually screened to identify additional eligible studies. Grey literature was searched through conference proceedings and dissertation databases (ProQuest). The reference list of included studies was also manually screened to identify additional studies relevant to the study objective.

Eligibility Criteria

Studies were included if they were cross-sectional studies, cohort studies or case-control studies reporting the prevalence of NAFLD in adult patients (≥18 years) with confirmed diagnosis of IBD (Crohn's disease, ulcerative colitis, or IBD-unclassified) based on standard clinical, endoscopic, radiological, and histological criteria, with NAFLD diagnosed by any method including imaging (ultrasound, computed tomography, magnetic resonance imaging, transient elastography), liver biopsy, or validated non-invasive biomarkers (e.g., Fatty Liver Index, Hepatic Steatosis Index), and providing sufficient data to calculate prevalence rates. Studies were excluded if they involved pediatric populations (<18 years), were case reports, case series, editorials, letters, or review articles, did not report NAFLD prevalence data or did not provide sufficient data for extraction, involved patients with other causes of liver disease including alcoholic liver disease, viral hepatitis, autoimmune hepatitis, primary biliary cholangitis, primary sclerosing cholangitis, hemochromatosis, Wilson's disease, or drug-induced liver injury, where NAFLD could not be distinguished from other hepatobiliary manifestations of IBD, or were duplicate publications (in cases of overlapping datasets, the study with the largest sample size or most comprehensive data was included).

Selection Process

Two independent reviewers screened all titles and abstracts identified through the literature search using predefined eligibility criteria. Full-text articles of potentially eligible studies were retrieved and assessed independently by the same reviewers. Any disagreements between reviewers were resolved through discussion or consultation with a third senior reviewer when consensus could not be reached. The study selection process was documented using a PRISMA (Preferred Reporting Items for Systematic Reviews and Meta-Analyses) flow diagram, indicating the number of records identified, screened, excluded with reasons, and finally included in the meta-analysis.

Data Extraction

Three reviewers independently extracted relevant information from each eligible study using a standardized data extraction sheet and then cross-checked the results. The data extracted included the last name of the first author, publication year, study location (country), and study design (cross-sectional, cohort, or case-control). Patient demographic and clinical characteristics extracted included the total sample size of the IBD cohort, number of subjects by sex (male and female), mean age, body mass index, and presence of metabolic syndrome components, including diabetes mellitus, hypertension, dyslipidemia, and obesity. IBD-specific variables extracted included IBD subtype (number of patients with Crohn's disease, ulcerative colitis, or IBD-unclassified), diagnostic criteria used for IBD diagnosis (clinical, endoscopic, radiological, and histological). Prevalence of NAFLD in IBD subjects. Discrepancies between reviewers regarding data extraction were resolved through discussion and consensus, with involvement of a senior fourth reviewer when necessary.

Quality Assessment

The methodological quality and risk of bias of included studies were assessed independently by two reviewers using appropriate tools based on study design. For cross-sectional and cohort studies, the Newcastle-Ottawa Scale (NOS) adapted for prevalence studies was used [16]. This scale evaluates three domains: selection of study groups (representativeness, sample size, non-respondents, ascertainment of exposure), comparability (control for confounding factors), and outcome assessment (assessment of outcome, statistical test). Studies were scored on a scale of 0-9 stars, with scores of 7-9 indicating high quality, 4-6 indicating moderate quality, and 0-3 indicating low quality.

Data Analysis

All statistical analyses were performed using R statistical software through the RStudio integrated development environment (RStudio, Boston, MA). The primary package used for meta-analysis was the 'meta' package. Extracted data from included studies were compiled into a structured dataset containing study identifiers, sample sizes, number of NAFLD cases, prevalence proportions, study design, IBD subtype, and publication year. Data were entered independently by two reviewers and cross-verified to ensure accuracy. The pooled prevalence of NAFLD in IBD patients was calculated using the random-effects model, accounting for expected heterogeneity across studies. Statistical heterogeneity among included studies was quantified using Cochran's Q test and the I² statistic. The I² statistic was interpreted as follows: 0-40% might not be important heterogeneity, 30-60% may represent moderate heterogeneity, 50-90% may represent substantial heterogeneity, and 75-100% represents considerable heterogeneity. Predefined subgroup analyses were conducted to explore sources of heterogeneity and examine prevalence differences across clinically relevant categories. Subgroups included study design (prospective cohort, retrospective cohort, cross-sectional), IBD subtype (Crohn's disease, ulcerative colitis), and year of publication (before 2020, 2020 onwards). Results were reported according to PRISMA guidelines, including pooled prevalence estimates with 95% confidence intervals, heterogeneity statistics, and results of subgroup analysis.

Results

**Figure 1:**
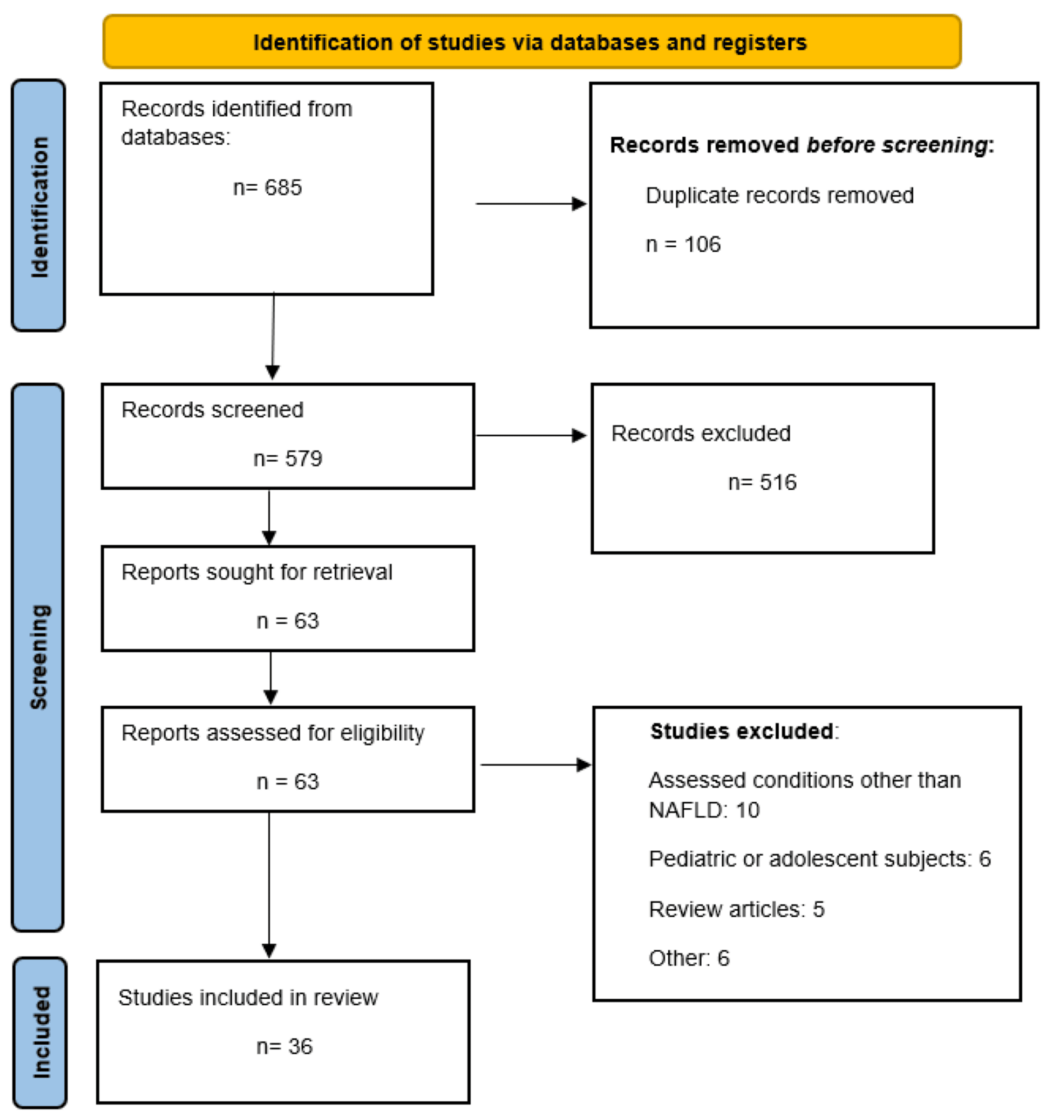
PRISMA flowchart (study selection process) PRISMA: Preferred Reporting for Systematic Reviews and Meta-Analysis.

Through online database searching, we identified 685 studies. After removing duplicate records, a total of 579 studies were initially screened using their titles and abstracts. After removing ineligible articles, 63 studies were thoroughly screened. Finally, 36 studies were included in this meta-analysis. Figure 1 shows the PRISMA flowchart of study selection.

Table 1 presents characteristics of included studies. A total of 36 studies were included in this analysis, encompassing diverse geographic regions across Europe (Italy, Spain, Germany, Croatia, Turkey, Romania), North America (United States, Canada), Asia (China, Japan, Korea, Taiwan), the Middle East (Qatar, Jordan), Africa (Egypt), and South America (Brazil). The majority of the studies were retrospective in design (n = 21), followed by cross-sectional (n = 9) and prospective (n = 6). Collectively, these studies included over 47 million individuals with inflammatory bowel disease (IBD), among whom a significant proportion were diagnosed with non-alcoholic fatty liver disease (NAFLD).

Diagnostic approaches for NAFLD varied widely among studies. The most used methods were hepatic ultrasonography, controlled attenuation parameter (CAP), hepatic steatosis index (HSI), fibrosis-4 index (FIB-4), and transient elastography. A few studies employed more advanced techniques such as magnetic resonance imaging-proton density fat fraction (MRI-PDFF), computed tomography (CT), or liver biopsy for confirmation. Several large-scale retrospective database studies from the United States contributed the majority of total participants, while smaller European and Asian studies provided detailed, clinically rich data with more standardized diagnostic assessments. Table 2 presents the quality assessment of the included studies.

**Table 1:** Included studies characteristics NAFLD: Non-Alcoholic Fatty Liver Disease; IBD: Inflammatory Bowel Disease; UC: Ulcerative Colitis; CD: Crohn's Disease; BMI: Body Mass Index; DM: Diabetes Mellitus; HTN: Hypertension; NR: Not Reported; CAP: Controlled Attenuation Parameter; HSI: Hepatic Steatosis Index; FIB-4: Fibrosis-4 Index; CT: Computed Tomography; MRI: Magnetic Resonance Imaging; VCTE: Vibration-Controlled Transient Elastography

Author	Year	Study Design	Region	Total subjects with IBD	Number of subjects with NAFLD	Diagnosis of NAFLD	UC (n)	CD (n)	Males (n)	Age	BMI	DM	HTN
Abenavoli et al. [17]	2024	Retrospective	Italy	272	64	Hepatic ultrasound	178	94	54	46	24	17	41
Abomhya et al. [18]	2022	Retrospective	United States	215049	5268	NR		215049	90860	53	1501	2186	1544
Almohannadi et al. [19]	2020	Retrospective	Qatar	913	108	NR	530	383	550	36.9	26.9	NR	NR
Balaban et al. [20]	2017	Prospective	Italy	36	11	CAP, HSI, or Ultrasound	17	19	17	43	25.3	2	NR
Bessissow et al. [21]	2016	Retrospective	Canada	321	108	HSI or FIB-4	NR	NR	151	33.7	NR	NR	NR
Bosch and Yeh [22]	2017	Retrospective	United States	118	32	Liver biopsy	NR	NR	25	47	27.6	5	9
Carrillo-Palau et al. [23]	2021	Cross-sectional	Spain	151	89	Abdominal ultrasonography	46	105	65	48	27	0	26
Castelhano et al. [24]	2025	Cross-sectional	Brazil	129	57	Hepatic ultrasound	67	62	40	NR	NR	16	31
Crispino et al. [25]	2022	Prospective	Italy	227	119	CAP, HSI	98	129	111	46.1	NR	NR	NR
Domislovic et al. [26]	2019	Retrospective	Croatia	250	101	HSI	83	167	130	40	NR	NR	NR
Elchert et al [27]	2018	Retrospective	United States	153,810	520	NR	NR	153,810	NR	NR	NR	NR	NR
Elchert et al. [28]	2018	Retrospective	United States	129300	370	NR	129300	NR	NR	NR	NR	NR	NR
Hoffmann et al. [29]	2020	Retrospective	Germany	455	213	Hepatic ultrasound	153	302	148	42	24.5	7	50
Hyun et al. [30]	2023	Retrospective	Korea	3356	560	HSI or FIB-4	2227	1129	1178	47.4	25.9	108	180
Kamel et al. [31]	2025	Cross-sectional	Egypt	81	19	CAP	24	57	40	33.27	24.08	6	3
Kang et al. [32]	2020	Retrospective	Korea	443	49	NR	NR	NR	NR	NR	NR	NR	NR
Kani et al. [33]	2019	Retrospective	Turkey	99	44	Transient elastography with CAP	39	58	58	45.59	NR	NR	NR
Lannone et al. [34]	2017	Prospective	Italy	378	106	Abdominal ultrasonography	NR	NR	NR	46.3	24.7	28	67
Li et al. [35]	2017	Retrospective	China	206	22	Hepatic ultrasound	69	137	133	40.4	19.65	NR	NR
Likhitsup et al. [36]	2019	Retrospective	United States	70	31	Computed tomography	13	57	31	38.6	26.6	8	NR
Lopes et al. [37]	2021	Cross-sectional	Brazil	71	32	Ultrasonography	37	34	NR	45.32	NR		NR
Magri et al. [38]	2019	Retrospective	Italy	178	72	Hepatic ultrasound	95	83	97	49.7	24.97	12	NR
Martínez-Domínguez et al. [39]	2024	Cross-sectional	Spain	741	332	Hepatic ultrasound	396	336	367	49	25.3	47	118
McHenry et al. [40]	2020	Cross-sectional	United States	311	118	Magnetic resonance proton density fat fraction	0	311	156	40	26	17	73
Onwuzo et al. [41]	2023	Retrospective	United States	46,667,720	34200	NR	119420	162620	21,504,950	NR	NR	16680	22420
Palumbo et al. [42]	2019	Prospective	Canada	384	126	Transient elastography	136	248	NR	NR	NR	NR	NR
Principi et al. [9]	2018	Cross-sectional	Italy	465	130	Hepatic ultrasound	NR	NR	NR	NR	NR	NR	NR
Ritaccio et al. [43]	2021	Retrospective	United States	1672	207	Imaging (ultrasound, CT, MRI) or liver biopsy	453	897	50.6	96	29.5	NR	NR
Rodriguez-Duque et al. [44]	2023	Cross-sectional	Spain	831	349	CAP	NR	NR	401	52	26.2	51	197
Sagami et al. [45]	2017	Retrospective	Japan	303	66	Transabdominal ultrasonography	0	303	226	36.9	19.8	NR	NR
Scrivo et al. [46]	2020	Prospective	Italy	208	43	Transient elastography, HSI	87	121	120	46.4	26	NR	NR
Simon et al. [47]	2018	Retrospective	United States	462	240	Liver attenuation	0	462	226	40	25.4	24	70
Tamimi et al. [48]	2025	Retrospective	Jordan	367	152	Hepatic ultrasound	194	173		NR	NR	NR	NR
Trifan et al. [49]	2022	Cross-sectional	Romania	82	38	VCTE and CAP	45	37	45	49	25.3	7	16
Veltkamp et al. [50]	2022	Retrospective	Germany	132	40	B-mode ultrasound, CAP, Hepatic fibrosis	53	79	57	42	23	NR	NR
Yen et al. [51]	2021	Retrospective	Taiwan	81	24	CAP	45	36	58	43.54	22.41	4	5

**Table 2:** Quality assessment of included studies

Author	Selection	Comparability	Outcome	Overall
Abenavoli et al. [17]	3	1	1	Fair
Abomhya et al. [18]	4	2	3	Good
Almohannadi et al. [19]	3	2	3	Good
Balaban et al. [20]	3	1	2	Fair
Bessissow et al. [21]	3	2	2	Good
Bosch and Yeh [22]	4	1	2	Good
Carrillo-Palau et al. [23]	4	2	2	Good
Castelhano et al [24]	3	1	2	Fair
Crispino et al. [25]	3	2	2	Good
Domislovic et al. [26]	3	2	2	Good
Elchert et al. [27]	3	1	2	Good
Elchert et al. [28]	3	1	2	Good
Hoffmann et al. [29]	4	2	3	Good
Hyun et al. [30]	4	2	3	Good
Kamel et al. [31]	4	1	2	Good
Kang et al. [32]	3	2	2	Good
Kani et al. [33]	4	1	2	Good
Lannone et al. [34]	3	1	2	Good
Li et al. [35]	3	2	2	Good
Likhitsup et al. [36]	3	2	2	Good
Lopes et al. [37]	2	1	2	Fair
Magri et al. [38]	4	2	2	Good
Martínez-Domínguez et al. [39]	4	2	3	Good
Mchenry et al. [40]	4	2	3	Good
Onwuzo et al. [41]	4	2	2	Good
Palumbo et al. [42]	3	1	2	Fair
Principi et al. [9]	3	2	2	Good
Ritaccio et al. [43]	4	1	2	Good
Rodriguez-Duque et al. [44]	3	2	2	Good
Sagami et al. [45]	4	2	3	Good
Scrivo et al. [46]	3	1	2	Fair
Simon et al. [47]	3	2	2	Good
Tamimi et al. [48]	3	1	2	Fair
Trifan et al. [49]	4	2	2	Good
Veltkamp et al. [50]	3	2	2	Good
Yen et al. [51]	3	2	3	Good

Prevalence of NAFLD in IBD Patients

**Figure 2:**
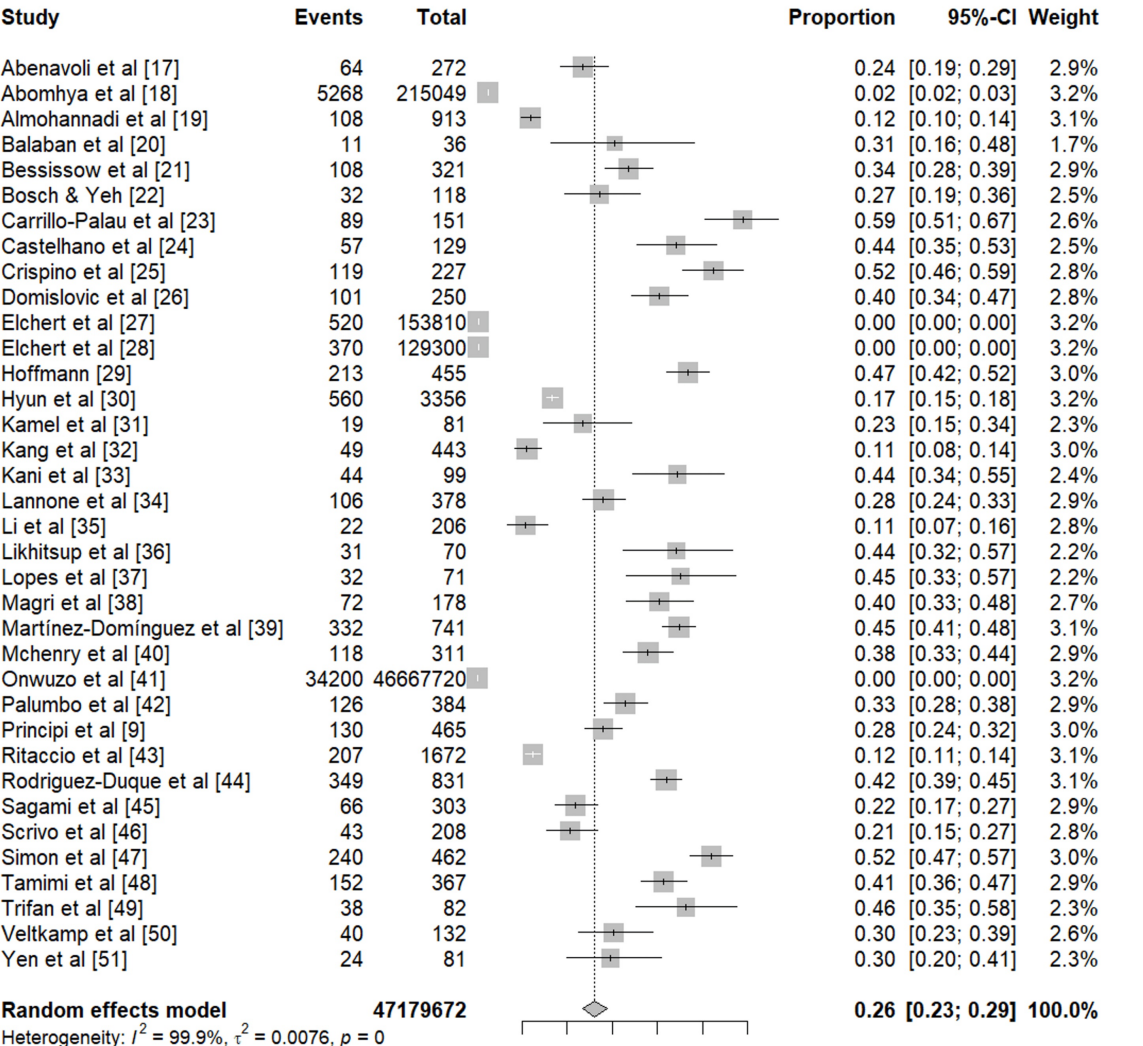
Forest plot (prevalence of NAFLD in IBD patients) References [9,17-51] NAFLD: Non-alcoholic fatty liver disease, IBD: Inflammatory bowel disease.

Figure 2 presents the forest plot showing the pooled prevalence of NAFLD in patients with IBD. A total of 36 studies comprising 47,179,672 patients with inflammatory bowel disease (IBD) were included in the meta-analysis to estimate the pooled prevalence of non-alcoholic fatty liver disease (NAFLD). The prevalence of NAFLD among IBD patients across individual studies ranged widely, from 0.01 to 0.59, reflecting substantial variation in study design, population characteristics, and diagnostic methods. Using a random-effects model, the pooled prevalence of NAFLD in patients with IBD was 26% (95% CI: 23-29%). There was considerable heterogeneity among the included studies (I² = 99.9%, τ² = 0.0076, p < 0.001), indicating that the true effect sizes varied substantially across studies. Despite this heterogeneity, the pooled estimate consistently demonstrated that approximately one in four patients with IBD had concurrent NAFLD, highlighting a significant comorbidity burden in this population.

Subgroup Analysis

We performed subgroup analysis based on study design (prospective cohort, retrospective cohort, and cross-sectional), type of IBD (UC and CD) and year of publication (before 2020 and 2020 onwards), and the results are presented in Table 3. Subgroup analysis revealed that the prevalence of NAFLD in patients with IBD varied according to study design, year of publication, and type of IBD. Retrospective studies reported a pooled prevalence of 19.5% (95% CI: 17-22%) with considerable heterogeneity (I² = 99.9%), whereas prospective studies showed a higher prevalence of 32.6% (95% CI: 22.7-43.4%; I² = 92.8%). Cross-sectional studies demonstrated the highest prevalence at 40.9% (95% CI: 35-47%; I² = 89.1%). When stratified by year of publication, studies published before 2020 reported a prevalence of 24.8% (95% CI: 20.9-28.9%; I² = 99.9%), compared with 27.5% (95% CI: 22.9-32.3%; I² = 99.7%) in studies published in 2020 or later. Regarding the type of IBD, ulcerative colitis (UC) patients had a prevalence of 17.0% (95% CI: 13.6-20.8%; I² = 99.6%), while Crohn’s disease (CD) patients had a higher prevalence of 26.1% (95% CI: 22.2-30.1%; I² = 99.9%). These findings suggest that study design, publication period, and IBD subtype influence the reported prevalence of NAFLD in this population.

**Table 3:** Subgroup analysis NAFLD: Non-alcoholic fatty liver disease, UC: Ulcerative colitis, CD: Crohn’s disease, CI: Confidence interval.

Subgroups	Study design	Prevalence	95% CI	I-Square
Study design	Retrospective	19.54	17 to 22	99.90%
	Prospective	32.64	22.7 to 43.41	92.80%
	Cross-sectional	40.93	35 to 47	89.10%
Year of Publication	Before 2020	24.79	20.87 to 28.94	99.90%
	2020 or onwards	27.49	22.89 to 32.34	99.70%
Type of IBD	UC	17.04	13.58 to 20.79	99.60%
	CD	26.05%	22.22 to 30.06	99.90%

Discussion

The present meta-analysis, encompassing 36 studies with over 47 million individuals with inflammatory bowel disease, demonstrates that approximately one in four patients with IBD (pooled prevalence: 26%, 95% CI: 23-29%) has concurrent non-alcoholic fatty liver disease. This finding underscores a substantial comorbidity burden in the IBD population and has important implications for clinical practice, screening strategies, and patient management. The observed prevalence is consistent with previous meta-analyses, though our updated analysis includes more recent studies and a significantly larger sample size, providing robust evidence for this association.

Our findings align closely with the meta-analysis by Zamani et al., which reported a pooled prevalence of NAFLD in IBD patients of 30.7% (95% CI: 26.5-34.9%) based on 44 studies with 14,947 patients [52]. Similarly, Lin et al. found a prevalence of 27.5% in their systematic review and meta-analysis [53]. The slight variations in prevalence estimates across meta-analyses can be attributed to differences in inclusion criteria, diagnostic methods for NAFLD, geographic distribution of included studies, and the time periods covered. Nevertheless, the consistency of findings across multiple independent meta-analyses strengthens the evidence that NAFLD is a common extraintestinal manifestation or comorbidity in patients with IBD. Earlier systematic reviews by Zou et al. [54] also reported similar prevalence rates, with their meta-analysis showing a pooled prevalence of 30.7% (95% CI: 26.5-34.9%) in IBD patients.

The comparison between IBD subtypes revealed that Crohn's disease patients had a higher prevalence of NAFLD (26.1%, 95% CI: 22.2-30.1%) compared to ulcerative colitis patients (17.0%, 95% CI: 13.6-20.8%). This differential prevalence has been noted in previous studies and may be explained by several mechanisms. The increased risk in CD patients may be related to disease-specific factors such as the higher frequency of small bowel involvement, which can lead to malabsorption and nutritional deficiencies, alterations in bile acid metabolism, and changes in gut microbiota composition. Additionally, CD patients may have higher rates of previous intestinal resections, particularly ileal resections, which have been associated with increased NAFLD risk due to disruption of the enterohepatic circulation and alterations in gut microbiota [55].

The pathophysiological mechanisms linking IBD and NAFLD are complex and multifactorial. Abenavoli et al. [17] highlighted the intricate interplay between gut microbiota dysbiosis, intestinal permeability, and liver disease in both conditions. Chronic systemic inflammation, a hallmark of IBD, plays a central role in the development and progression of NAFLD. Pro-inflammatory cytokines such as tumor necrosis factor-alpha (TNF-α), interleukin-6 (IL-6), and interleukin-1β (IL-1β) are elevated in IBD patients and can promote hepatic steatosis through multiple mechanisms, including increased hepatic lipogenesis, decreased fatty acid oxidation, and insulin resistance. The translocation of bacterial products through the compromised intestinal barrier in IBD patients may further contribute to hepatic inflammation through activation of Kupffer cells and subsequent production of reactive oxygen species. Rojas-Feria et al. comprehensively reviewed the hepatobiliary manifestations in inflammatory bowel disease, emphasizing the complex interactions between the gut, drugs, and the liver in these patients [56].

Several studies have demonstrated gut dysbiosis in both NAFLD and IBD, with distinct but overlapping microbial signatures. Fujimoto et al. [57] reported decreased abundance of Faecalibacterium prausnitzii in the gut microbiota of Crohn's disease patients, while Iino et al. [58] found a significant decrease in Faecalibacterium species in patients with NAFLD in a large BMI- and sex-matched population study. Andoh et al. compared fecal microbiota profiles between ulcerative colitis and Crohn's disease using terminal restriction fragment length polymorphism analysis, revealing distinct microbial patterns [59]. Jiang et al. demonstrated that dysbiosis of gut microbiota is associated with inflammation and impaired mucosal immune function in the intestine of humans with NAFLD [60].

Traditional metabolic risk factors, including obesity, type 2 diabetes mellitus, hypertension, and dyslipidemia, remain important contributors to NAFLD in IBD patients. Onwuzo et al. [41] demonstrated that obesity conferred the highest risk for NASH development (OR: 6.10; 95% CI: 5.93-6.27%), followed by type 2 diabetes (OR: 3.08; 95% CI: 3.00-3.17%) and hyperlipidemia (OR: 3.03; 95% CI: 2.94-3.12%). These findings underscore the importance of addressing modifiable metabolic risk factors in IBD patients to reduce NAFLD risk. Souza et al. examined metabolic syndrome and risk factors for NAFLD, demonstrating strong associations between these conditions [61]. The global increase in obesity and metabolic syndrome, coupled with the rising incidence of IBD, particularly in newly industrialized countries undergoing lifestyle transitions, creates a "perfect storm" for increasing NAFLD prevalence in IBD populations. Younossi et al. provided a comprehensive meta-analytic assessment of the global epidemiology of NAFLD, reporting a global prevalence of 25.24% (95% CI: 22.10-28.65%) in the general population, which serves as an important comparator for our findings in IBD patients [1].

The substantial heterogeneity observed across studies (I² = 99.9%) warrants careful interpretation of pooled estimates. This heterogeneity likely reflects differences in diagnostic criteria for NAFLD, study populations (age, ethnicity, disease duration), geographic variations in metabolic risk factor prevalence, and differences in IBD disease characteristics and treatment patterns. The wide range of diagnostic methods employed across studies, from simple ultrasonography to advanced techniques such as MRI-PDFF and liver biopsy, contributes to this variability.

Clinical implications of our findings are significant. Given that approximately one in four IBD patients has NAFLD, there is a strong rationale for implementing systematic screening protocols in this population. Early detection of NAFLD and hepatic fibrosis is crucial, as progression to advanced fibrosis and cirrhosis can occur, particularly in patients with multiple metabolic risk factors. Wong et al. highlighted that NASH is the second leading cause of liver transplantation in the United States, emphasizing the clinical importance of early identification and intervention [62]. Non-invasive assessment tools such as CAP, HSI, FIB-4 score, and transient elastography should be considered for routine screening in IBD patients, especially those with traditional risk factors for NAFLD.

Our study has several limitations that warrant consideration. The substantial heterogeneity across included studies limits the precision of pooled estimates and suggests caution in generalization. The predominance of retrospective studies may introduce selection bias and underestimate true prevalence. Variations in NAFLD diagnostic methods across studies affect the comparability of results. Finally, most included studies did not assess the severity of hepatic steatosis or the presence of advanced fibrosis, limiting our understanding of clinically significant liver disease burden in this population. Future studies should employ standardized diagnostic criteria, include longitudinal follow-up to assess disease progression, and evaluate the impact of specific IBD treatments on NAFLD development and progression.

## Conclusions

This comprehensive meta-analysis of 36 studies encompassing over 47 million IBD patients demonstrates that approximately one in four individuals with inflammatory bowel disease has concurrent non-alcoholic fatty liver disease, with a pooled prevalence of 26%. The significantly higher prevalence in Crohn's disease patients compared to ulcerative colitis patients, along with variations across study designs and diagnostic methods, highlights the complex interplay between gut inflammation, dysbiosis, metabolic factors, and hepatic steatosis. These findings underscore the critical importance of implementing systematic NAFLD screening protocols in IBD populations, particularly for patients with traditional metabolic risk factors. The substantial comorbidity burden necessitates integrated multidisciplinary care approaches addressing both gastrointestinal and hepatic manifestations. Future research should focus on standardized diagnostic criteria, longitudinal disease progression assessment, and evaluation of IBD-specific treatment impacts on NAFLD development and outcomes.
